# 2023 European Society of Cardiology guidelines for the management of infective endocarditis: Statement of endorsement by the NVVC *Short version*

**DOI:** 10.1007/s12471-025-02011-9

**Published:** 2025-12-18

**Authors:** Annelot J. L. Peijster, Cees van Nieuwkoop, Ruud W. M. Keunen, Susanne Felix, Berend J. van Welzen, Ilse J. E. Kouijzer, C. H. Edwin Boel, Nelianne J. Verkaik, Ka Yan Lam, Robert J. M. Klautz, Andor W. J. M. Glaudemans, Ricardo P. J. Budde, Alexander H. Maass, Reinoud E. Knops, Otto Kamp, Wilco Tanis

**Affiliations:** 1 Department of Cardiology, Amsterdam University Medical Centre, Amsterdam, The Netherlands; 2 Department of Cardiology, Haga Teaching Hospital, The Hague, The Netherlands; 3 Department of Internal Medicine, Haga Teaching Hospital, The Hague, The Netherlands; 4 Department of Neurology, Haga Teaching Hospital, The Hague, The Netherlands; 5 Department of Cardiology, Catharina Hospital, Eindhoven, The Netherlands; 6 Department of Infectious Diseases, University Medical Centre Utrecht, Utrecht, The Netherlands; 7 Department of Internal Medicine, Radboud University Medical Centre, Nijmegen, The Netherlands; 8 Department of Medical Microbiology, University Medical Centre Utrecht, Utrecht, The Netherlands; 9 Department of Microbiology, Erasmus Medical Centre, Rotterdam, The Netherlands; 10 Department of Cardiothoracic Surgery, Catharina Hospital, Eindhoven, The Netherlands; 11 Department of Cardiothoracic Surgery, Amsterdam University Medical Centre, Amsterdam, The Netherlands; 12 Department of Cardiothoracic Surgery, Leiden University Medical Centre, Leiden, The Netherlands; 13 Department of Nuclear Medicine and Molecular Imaging, University Medical Centre Groningen, Groningen, The Netherlands; 14 Department of Radiology & Nuclear Medicine, Erasmus Medical Centre, Cardiovascular Institute, Rotterdam, The Netherlands; 15 Department of Cardiology, University of Groningen, University Medical Centre Groningen, Groningen, The Netherlands

**Keywords:** Infective endocarditis, Infection, Endocarditis Team, Prevention, Cardiac imaging, Diagnostic criteria, Microbiological diagnosis, Prosthetic valve endocarditis, Device endocarditis, Cardiac surgery

## Abstract

This review evaluates the 2023 European Society of Cardiology (ESC) guidelines for the management of infective endocarditis (IE) and offers insights into topics particularly relevant to clinical practice in the Netherlands. The multidisciplinary IE Working Group assessed all ESC recommendations and concluded that the majority could be endorsed, albeit with certain modifications. The IE Working Group presents a refined (and simplified) antibiotic prophylactic regimen and an updated table to guide the analysis of suspected blood culture-negative IE. Furthermore, the pivotal role of the Endocarditis Team in the management of patients with suspected and confirmed IE was reaffirmed, along with reinforced recommendations for nearly all cardiac and extracardiac imaging in these cases. Most notable, a preliminary recommendation was issued for switching to oral antibiotic therapy in patients with native valve endocarditis caused by viridans streptococci, while awaiting the revision of the Dutch Working Group on Antibiotic Policy (SWAB) IE guideline. In addition, the surgical recommendations were evaluated and revised, including improved clinical criteria in case of cardiac surgery following neurological complications of IE and an advised disregard of the new class I ESC recommendation for surgery in early prosthetic valve endocarditis (< 6 months). Moreover, an additional device recommendation was proposed for the choice of (alternate) devices in case of device reimplantation after IE.

**Supplementary Information:**

The online version of this article (10.1007/s12471-025-02011-9) contains supplementary material, which is available to authorized users.

## Introduction

In October 2023, a new European Society of Cardiology (ESC) guideline for the management of infective endocarditis (IE) was released, updating the 2015 guideline [1, 2]. A multidisciplinary IE Working Group was initiated by the Dutch Society of Cardiology (Nederlandse Vereniging voor Cardiologie, NVVC) to review how the 2023 ESC guidelines for IE management could be effectively implemented in the Netherlands. A key distinction of the IE Working Group, compared to the ESC Task Force, is the inclusion of clinical microbiologists and a neurologist, along with an overall balanced representation of various medical specialists. The new guideline recommendations focus on prevention, diagnostic criteria, multimodality imaging, oral antibiotic therapy, surgical indications, and timing [1]. Besides these main themes, this guideline also reinforced their recommendation of the Endocarditis Team (with an upgrade from class IIa, level B to class I, level B) and highlights patient-centered care and shared decision-making in IE. The IE Working Group supports these recommendations as the Endocarditis Team enhances evidence-based practice by integrating guideline and evidence discussions, individual (clinical) patient information, and the experience and expertise of all involved specialists. And while the emphasis on patient-centered care and shared-decision making is always important in healthcare, the IE Working Group confirms that the heterogeneity, severity, complexity, and prolonged treatment duration of IE should underscore their significance. The new ESC guideline also includes a new IE education card for high-risk patients to help prevent IE. Certainly, patient education and awareness are strongly recommended by the IE Working Group. Therefore, along with this endorsement paper, a patient IE card (including QR-code to facilitate sharing and digital storing) will be introduced and distributed for use in the Netherlands (Fig S1 in the Supplementary Appendix). Before further evaluating the guideline, it should be noted that most recommendations of this specific ESC guideline rely on low-level evidence; with 56% of the recommendations based on level C evidence and only 3% supported by level A evidence [1, 3]. This paper summarises the recommendations of the IE Working Group, proposing several adjustments to the ESC guideline, at least within the Dutch context. However, this article represents a condensed version of the full IE endorsement paper (10.1007/s12471-025-02010-w), summarising only selected aspects most relevant for cardiology and cardiac surgery. Readers are referred to the full version for comprehensive multidisciplinary coverage.

## Imaging

In IE multimodality imaging, the use of cardiac computed tomography (CT), positron emission tomography and computed tomography (PET/CT), as well as cerebral magnetic resonance imaging (MRI), has led to major improvements in diagnostics (especially of prosthetic valve endocarditis (PVE)) [1, 4–6]. The new guideline reinforces and further upgrades most indications for all cardiac and extracardiac imaging for (suspected) IE patients. While the IE Working Group endorses the majority of the new imaging recommendations, it remains crucial (and may be regarded as self-evident) to employ these imaging techniques only when the results offer clinically relevant information and/or a potential change in the management of IE.

**Fig. 1:**
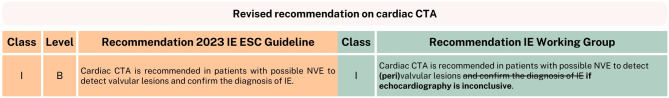
Revised recommendations on cardiac CTA. *CTA* computed tomography angiography, *IE* infective endocarditis, *ESC* European Society of Cardiology, *NVE* native valve endocarditis. *This figure is included in the condensed version; additional figures are available in the full version* (10.1007/s12471-025-02010-w)

The guideline updated and upgraded its recommendation on transoesophageal echocardiogram (TOE) to a class I, level C indication for TOE ‘in patients with suspected IE, even in cases with positive transthoracic echocardiogram (TTE), except in isolated right-sided native valve IE with good quality TTE examination and unequivocal echocardiographic findings’. The IE Working Group endorses this recommendation, as it will stimulate consideration of a TOE more quickly, and in all IE-related cases; however, we also emphasize that no new evidence supports this upgrade. Given that it is a semi-invasive procedure, the clinical implications should be carefully considered, particularly in cases that are ineligible for surgery and require antibiotic treatment similar to that for IE, regardless of the TOE [7]. The new class I, level B recommendation for ‘cardiac computed tomography angiography (CTA) in patients with possible native valve endocarditis (NVE) to detect valvular lesions and confirm the diagnosis of IE’ is changed by the IE Working Group by stating (peri)valvular instead of valvular, adding ‘if echocardiography is inconclusive’ and deleting ‘confirm the diagnosis of IE’ (Fig. 1; [8]). Although CTA may offer somewhat limited additional value in this patient group with possible NVE, we agree it can be useful to have the option to perform a CTA when suspicion of IE persists (and echocardiography yielded negative or inconclusive results). It should be noted that performing a CTA in patients with possible NVE may lead to a negative result, which does not rule out endocarditis, as vegetations may go undetected on CTA.

## Diagnostic criteria

In addition to the new ESC guideline featuring updated major and minor criteria for the diagnosis of IE, the revised modified Duke criteria, the Duke-International Society for Cardiovascular Infectious Disease criteria (Duke-ISCVID), were also published last year [9]. Both new criteria have shown to increase sensitivity compared to the 2000 modified Duke criteria and the 2015 ESC criteria; however, the Duke-ISCVID appear to preserve specificity better than the 2023 ESC criteria [10–12]. According to both these diagnostic tools, the diagnosis of IE is considered definite when two major criteria or one major and three minor criteria are met. In rare cases, five minor criteria without any major criteria may also be regarded as definite IE.

**Fig. 2:**
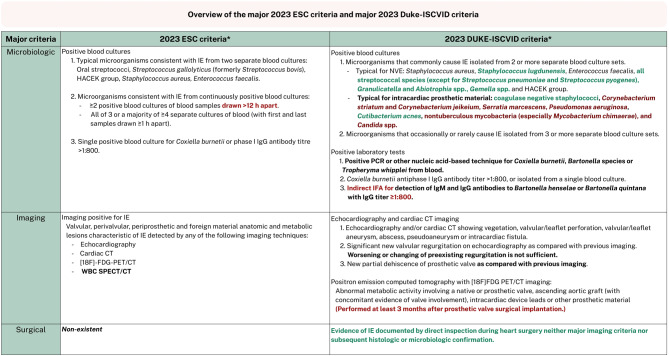
Overview of the major 2023 ESC criteria and major 2023 Duke-ISCVID criteria [1, 9]. *ESC* European Society of Cardiology, *Duke-ISCVID* Duke—International Society for Cardiovascular Infectious Diseases Criteria, *HACEK group* *Haemophilus parainfluenzae*, *Haemophilus aphrophilus*, *Aggregatibacter actinomycetemcomitans*, *Cardiobacterium hominis*, *Eikenella corrodens*, and *Kingella kingae*, *h* hour, *NVE* native valve endocarditis, *PCR* polymerase chain reaction, *IgM* Immunoglobulin M, *IgG* Immunoglobulin G, *CT* computed tomography, *[18F]-FDG-PET/CT* 18F-Fluorodeoxyglucose positron-emission tomography/computed tomography, *WBC SPECT/CT* white blood cells single photon emission computed tomography/computed tomography. *Please note, this table provides a concise overview and not the full published text. The main differences between the two criteria are highlighted in bold, while agreement or disagreement by the IE Working Group is indicated in green and red, respectively, as detailed in the text. *This figure is included in the condensed version; additional figures are available in the full version* (10.1007/s12471-025-02010-w)

The IE Working Group has no comments on the minor ESC criteria. However, it generally favors the major criteria of the Duke-ISCVID criteria, being more applicable in practice (e.g., less stringent timing for blood culture collection [13] and the inclusion of causative pathogens specific to PVE). The Duke-ISCVID criteria comprise three major criteria, compared to two in the new ESC criteria (Fig. 2). First, the microbiologic major criteria include a more extensive list of causative pathogens marked as typical, especially with the addition of typical bacteria for intracardiac prosthetic material. While the IE Working Group welcomes this change (acknowledging the prevalence of PVE), we recommend limiting it to typical agents for infection of prosthetic valves as opposed to all intracardiac prosthetic material (e.g., left ventricular assist devices) and therefore retaining only *Cutibacterium acnes* and coagulase-negative staphylococci as typical causative pathogens [14]. The other pathogens are less associated with IE, which also aligns with the current standard antibiotic treatment for PVE [1, 9, 15]. Moreover, we propose to revise the microbiologic criterion for ‘indirect immunofluorescence assays (IFA) for detection of IgM and IgG antibodies to *Bartonella henselae* or *Bartonella quintana* with immunoglobulin G (IgG) titer ≥ 1:800’ to retain a ‘high antibody titer to *Bartonella henselae* or *Bartonella quintana*’, since IFA is not always available. The preferred method may vary between microbiologic laboratories; in the Netherlands, Enzyme Linked Immune Sorbent Assay (ELISA) or Indirect chemiluminescent immunoassay (CLIA) are more reflective of standard practice. Since the cut-off is not well defined for tests other than IFA, a Bartonella PCR should be performed in addition to serology in case of blood-culture negative IE (Tab 1). Second, while we suggest that the imaging major criteria from the new ESC guideline may be utilized, it is important to clearly delineate the differences. The ESC imaging criteria has become open to broad interpretation as it states to be present with any ‘valvular, perivalvular/periprosthetic and foreign material anatomic and metabolic lesions characteristic of IE detected by echocardiography, cardiac computed tomography, 18F-Fluorodeoxyglucose positron-emission tomography/computed tomography (FDG-PET/CT) or white blood cell single photon emission computed tomography (WBC SPECT/CT)’. This differs from both the 2015 ESC guideline and the Duke-ISCVID criteria, which are more specific in naming the potential findings, such as vegetation or abscess. As it is less precise, it also omits the note that FDG-PET/CT should only be performed at least 3 months after prosthetic valve implantation in order to provide a major criterion (while within 3 months could result in a minor criterion) [1, 5, 9]. The IE Working Group agrees that the 3‑month timing of FDG-PET/CT can be disregarded, but for appropriate interpretation, the timing of the FDG-PET/CT after prosthetic valve implantation should be considered. Although this issue remains debated, we believe it is important to take into account the timing in combination with the surgical technique, the surgical materials used, and the uptake pattern of the FDG-PET/CT, following multiple studies including the TEPvENDO study as well as a Dutch multicenter study [16–19]. Moreover, consultation with an experienced specialist is strongly recommended for proper assessment of the FDG-PET/CT scan, particularly in this population. Besides this remark, the IE Working Group does agree with the new, broader imaging criteria of the ESC guideline, while stressing to be more aware of the fact that the assessment now is increasingly dependent on the individual(s) interpreting the results. Third, the new major criterion, exclusive to the Duke-ISCVID criteria, is the surgical criterion, which indicates being positive when there is documented evidence of IE observed during inspection in cardiothoracic surgery, without the need for confirmation of imaging, histology, or microbiology. The IE Working Group believes that the surgeon’s observations should constitute as a major criterion, especially since the patient then has a new prosthetic valve in (or near) the area where signs of IE were observed.

## Surgical indication

**Fig. 3:**
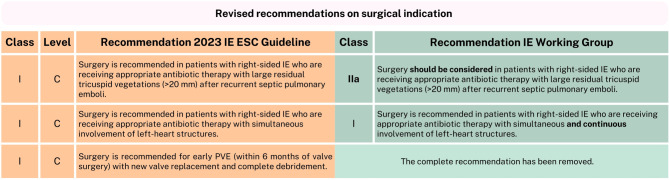
Revised recommendations on surgical indication. *IE* infective endocarditis, *ESC* European Society of Cardiology, *mm* millimeter, *PVE* prosthetic valve endocarditis. *This figure is included in the condensed version; additional figures are available in the full version* (10.1007/s12471-025-02010-w)

The three main categories that indicate surgery in IE patients remain heart failure, uncontrolled infection, and prevention of embolic events [1]. The IE Working Group has endorsed most of the new guidelines’ surgical recommendations. However, there are some remarks regarding one left-sided IE recommendation, two right-sided IE recommendations, and the new recommendation for early PVE (Fig. 3). In addition, the new guideline introduced a general change in the timing of performing surgery for IE. Urgent surgery is now classified as within 3–5 days, as well as leaving it to the discretion of the Endocarditis Team (while not delaying surgery by waiting on the Endocarditis Team) [1]. We endorse this change in surgical timing, as the trend in most studies has been to perform surgery quickly in order to reduce mortality, most of all in patients with heart failure or prevention of an embolism as the surgical indication [20–24].

Another general change since the 2015 guideline is the adoption of a different classification for vegetation length. The previous guideline made a distinction between vegetations > 10 millimeters (mm), > 15 mm and > 30 mm in their surgical recommendations, while the 2023 guideline only distinguishes between vegetations >10 mm and those smaller for formal surgical recommendations [1, 2]. Although most (limited) studies seem to suggest that early surgery may benefit patients who are at higher risk of embolization, the available data on this, including specific vegetation sizes, remain insufficient [20, 22, 23, 25, 26]. Individualized decision-making is therefore recommended for this (now to a greater degree) heterogeneous group. In line with this, the new IIb recommendation for urgent surgery in left-sided IE with a vegetation ≥ 10 mm, without severe valve dysfunction or without clinical evidence of embolism and low surgical risk, was evaluated. The IE Working Group endorses this recommendation because it addresses this heterogeneous group, and class IIb allows for the flexibility needed. For this patient group, it remains unclear whether surgery or conservative treatment is more effective. The ongoing randomized controlled ASTERIx trial may provide clarity in the future [27]. As for the new right-sided IE surgical indications, the IE Working Group could endorse most of them. However, two specific recommendations warrant further consideration. The first is the new class I recommendation for surgery in patients with right-sided IE who are receiving appropriate antibiotic therapy with large residual tricuspid vegetations (> 20 mm) after recurrent septic pulmonary emboli. We suggest allowing some flexibility (which a class I indication does not), as none of the presented evidence supports this recommendation [25, 28]. A IIa indication would allow deviation, for example, in cases where patients lack relevant tricuspid valve insufficiency and because emboli are generally less alarming from a right-sided source [29].

The second new class I indicated for surgery in patients with right-sided IE to review, is that of patients with simultaneous involvement of left-heart structures. The IE Working Group specifies this recommendation to ‘simultaneous and continuous involvement’ [1]. The IE Working Group does not consider a class I indication appropriate for patients with solely left- and right-sided endocarditis (without continuous involvement and) without other surgical indication(s).

Finally, a new class I, level C recommendation was made by the new guideline, which recommends surgery for early PVE (within 6 months of valve surgery) with new valve replacement and complete debridement [1]. The first year after implantation is considered a vulnerable period due to increased healthcare contact and still ongoing endothelialisation of the prosthetic valve. Moreover, the in-hospital mortality rate seems to be higher in the early PVE group as opposed to patients with PVE later after implantation [30, 31]. Given this knowledge, we consider the new focus on this high-risk group (early PVE) in the guideline appropriate. However, there is no supporting evidence for the specific recommendation itself, much less a class I indication.

## Cardiac implantable electronic devices

**Fig. 4:**
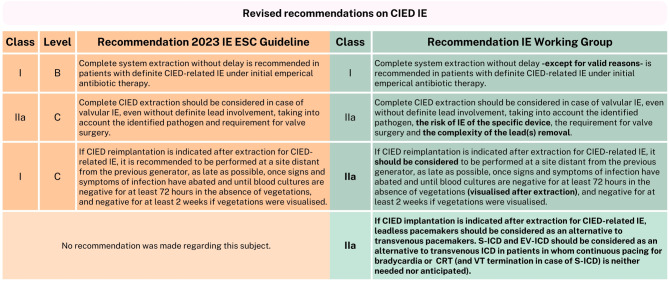
Revised recommendations on CIED IE. *IE* , infective endocarditis, *ESC* European Society of Cardiology, *CIED* cardiac implantable electronic device, *S‑ICD* subcutaneous implantable cardioverter-defibrillator, EV-ICD, extravascular implantable cardioverter defibrillator, *ICD* implantable cardioverter defibrillator, *VT* ventricular tachycardia, *CRT* cardiac resynchronization therapy. *This figure is included in the condensed version; additional figures are available in the full version* (10.1007/s12471-025-02010-w)

The IE Working Group endorses nearly all CIED-related recommendations; however, there are some considerations and comments, and one new proposed recommendation (Fig. 4).

The new class IIa, level C recommendation, that ‘immediate epicardial pacemaker implantation should be considered in patients undergoing surgery for valvular IE and complete atrioventricular block if one of the established predictors is present’ is endorsed, as the opportunity naturally presents itself during thoracotomy and the procedure is straightforward, safe and potentially reduces the delay to another pacemaker implantation [1, 32]. In addition, the class IIa indication leaves flexibility to adjust this treatment approach if it’s not desirable in an individual patient case. Moreover, it is worth noting that a leadless pacemaker can also be implanted during surgery as well as peri-operative in collaboration with an experienced electrophysiologist [33–35]. As for the new class I, level B indication that ‘complete system extraction is recommended without delay in patients with definite CIED-related IE’, there are some aspects to reflect on. The trend towards earlier extraction is understandable as there is no rationale to wait, like with PVE (the theory to treat with antibiotics for some time before intervention) [36, 37]. However, ‘without delay’ should not necessarily be perceived as an urgent indication. In some cases, there may be reasons to wait, as percutaneous extraction is usually preferred over surgical; however large vegetations or hemodynamic instability may make percutaneous extraction impossible at the initial stage. Lastly, although this is a class I recommendation, in practice, this specific recommendation will require more careful consideration and shared decision-making, as frailty, comorbidities, and high extraction risk are common in this population, and there are cases where conservative treatment is effective [38].

In the area of CIED extraction, the 2015 class IIb, level C recommendation that ‘complete CIED extraction may be considered in case of valvular IE and an intracardiac device with no evidence of device infection’, was upgraded to class IIa, level C and nuanced by the addition ‘to take into account the identified pathogen and requirement for valve surgery’ [1, 2]. We propose to further extend the recommendation to also take into account the complexity of the lead(s) removal, considering factors such as the age of the leads and the presence of abandoned leads, as well as the risk of IE of the specific device, for example, weighing the lower risk of IE for leadless pacemakers [39–43]. The 2015 class IIa, level C indication for complete hardware removal on the basis of occult bacteraemia without another apparent source of infection, was refined with the addition of ‘in case of persistent/relapsing bacteraemia after a course of antimicrobial therapy’ and the recommendation was split into a IIa, level C recommendation for occult Gram-positive bacteraemia or fungaemia and a class IIb, level C, recommendation for occult Gram-negative bacteraemia [1, 2]. We endorse these changes, stressing the need to consider the severity of the causative pathogen and the associated risk of IE [44–47].

Another revised and upgraded in class (IIa to I) recommendation was issued, regarding reimplantation and its timing. The recommendation states: ‘if CIED reimplantation is indicated after extraction for CIED-related IE, it is recommended to be performed at a site distant from the previous generator, as late as possible, once signs and symptoms of infection have abated and until blood cultures are negative for at least 72 h in the absence of vegetations, and negative for at least 2 weeks if vegetations were visualised’. There is still insufficient evidence for the best timing of reimplantation after CIED IE [48]. This new recommendation provides more direction for the timing of reimplantation than before, which in practice, is appreciated by the treating physician. However, we would change the recommendation to class IIa, factoring in the lack of supporting evidence for the reported timing [49, 50]. Additionally, we would clarify the term ‘vegetations’, as they specifically imply vegetations found after extraction (also called ‘ghosts’), which have been associated with mortality and reinfection [51].

Finally, the new guideline remains conservative in its recommendations regarding device choice for reimplantation following CIED-related IE. The options for alternative devices, such as leadless pacemakers and subcutaneous implantable cardioverter defibrillators (S-ICD’s), are mentioned, but no formal recommendations were made regarding them. In addition, the extravascular implantable cardioverter defibrillator (EV-ICD) is also a valid option for many implantable cardioverter defibrillator carriers [52]. We advocate that these options should be considered as alternatives to transvenous systems, citing increased clinical experience, reduced infection risk demonstrated by results from various studies, such as the Micra postapproval registry, along with support from other established and widely used guidelines [53–58]. The ESC guideline on cardiac pacing and cardiac resynchronization therapy recommends (class IIa, level B) implanting leadless pacemakers in patients with previous infection and the American guideline for management of patients with ventricular arrhythmias and the prevention of sudden cardiac death includes the (class I) recommendation for S‑ICD implantation in patients at high risk of infection. It is important to note that appropriate patient selection remains key, taking into account all patient-specific circumstances [53, 57, 59].

### Full version IE endorsement paper

**Fig. 5:**
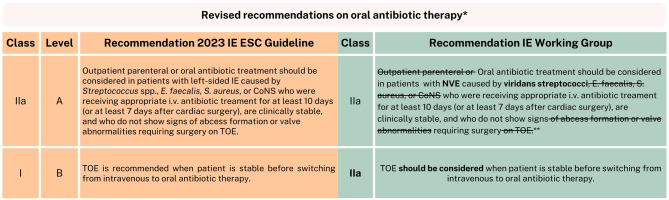
Revised recommendations on oral antibiotic therapy. *IE* infective endocarditis, *ESC* European Society of Cardiology, *E.* *faecalis* *Enterococcus faecalis,* *S.* *aureus* *Staphylococcus aureus*, *CoNS* coagulase-negative staphylococci, *i.v.* intravenous, *TOE* transoesophageal echocardiogram, * Please note, these revised recommendations are preliminary, in anticipation of the new IE Dutch Working Group on Antibiotic Policy (Stichting Werkgroep Antibiotica Beleid, SWAB) guideline. ** The by IE Working Group recommended oral antibiotic therapy for this population (NVE caused by *viridans* streptococci) is amoxicillin 1000 milligrams 4 times/day (with a minimum inhibitory concentration for penicillin of < 0.25 μg/mL)

In addition to the topics discussed above, the complete version of the IE endorsement paper further elaborates on several new and clinically relevant recommendations. Among these, the updated approach for oral antibiotic therapy in patients with native valve endocarditis caused by viridans streptococci deserves special attention. This novel recommendation is briefly illustrated in Fig. 5, while the complete rationale and implications are described in detail in the full version (10.1007/s12471-025-02010-w).

## Conclusion

The multidisciplinary IE Working Group reviewed all 2023 ESC recommendations on the management of IE and concluded that most could be endorsed. However, specific modifications are recommended to more accurately reflect the strength of the underlying evidence and to better align with the national context. Key adjustments include a simplified antibiotic prophylaxis regimen, revised diagnostic, surgical, and device recommendations and an adjusted recommendation for oral antibiotic therapy, in line with the anticipated revised Dutch Working Group on Antibiotic Policy (SWAB) IE guideline. This review provides guidance for IE management within the Dutch clinical setting, albeit in a condensed format. For comprehensive multidisciplinary coverage, readers are referred to the complete online version (10.1007/s12471-025-02010-w).

## Supplementary Information

*Patient education*
Please note that a physical version of the patient information card will be distributed nationally as widely as feasible. Conveniently, the inclusion of the QR code enables immediate and easy digital storage and sharing, ensuring broad accessibility. For English-speaking patients, we refer to the patient education card provided in the 2023 ESC guidelines on the management of infective endocarditis.
Fig S1. Dutch patient information card to prevent infective endocarditis in patients at high risk.
