# Supplementary material for: 2023 European Society of Cardiology guidelines for the management of infective endocarditis: Statement of endorsement by the NVVC Short version
Source: Neth Heart J. 2025 Dec 18;34(1):7–17. doi: 10.1007/s12471-025-02011-9 (PMC12779872; doi:10.1007/s12471-025-02011-9)
Supplement: Supplementary file 1 — Patient education Please note that a physical version of the patient information card will be distributed nationally as widely as feasible. Conveniently, the inclusion of the QR code enables immediate and easy digital storage and sharing, ensuring broad accessibility. For English-speaking patients, we refer to the patient education card provided in the 2023 ESC guidelines on the management of infective endocarditis. Fig S1. Dutch patient information card to prevent infective endocarditis in patients at high risk. [file 12471_2025_2011_MOESM1_ESM.pdf]

Patiëntenkaart: voorkom endocarditis bij verhoogd risico

Wat is endocarditis?

Endocarditis is een infectie (ontsteking) aan de binnenkant van het hart, meestal van de hartkleppen. De ziekte komt niet vaak voor, maar sommige mensen hebben een verhoogd risico. Dit geldt bijvoorbeeld voor mensen met een kunsthartklep, bepaalde aangeboren hartafwijkingen en mensen die al eerder endocarditis hebben gehad.

Wat is extra belangrijk voor mensen met een hoger risico?

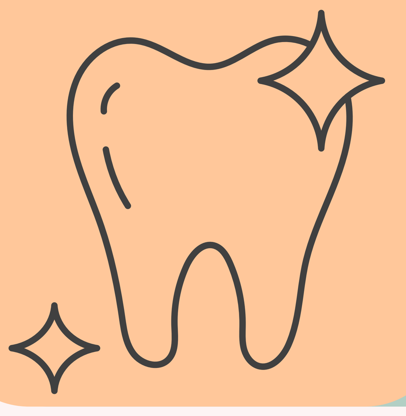

Goede mondhygiëne

Dagelijks tandenpoetsen ('s ochtends en 's avonds), dagelijks flossen en regelmatige afspraken bij de tandarts.

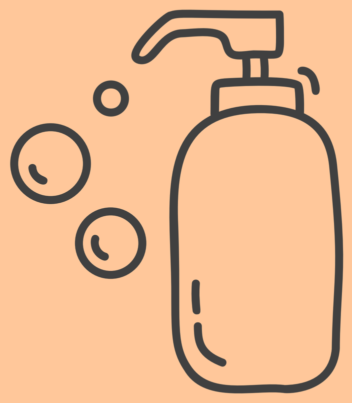

Goede huidhygiëne

Voorkom wondjes van de huid en vermijd tatoeages en piercings. Let in het geval van een verwonding goed op tekenen van infectie (zoals roodheid, zwelling, gevoeligheid of pus).

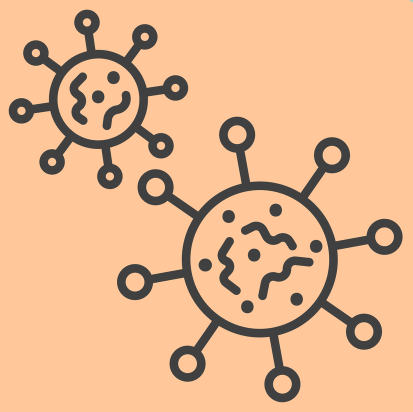

Wees alert op tekenen van infectie zoals koorts

Neem bij koorts of koude rillingen zonder duidelijke oorzaak (bijvoorbeeld zonder andere griepverschijnselen) altijd contact op met de huisarts. Neem ook altijd opnieuw contact op als de koorts aanhoudt of terugkomt.

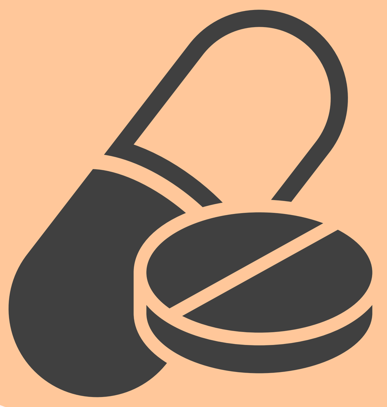

Gebruik niet zomaar antibiotica

Gebruik alleen antibiotica als een dokter dit voorschrijft. Zorg er ook voor dat de antibioticakuur altijd wordt afgemaakt, ook als er geen klachten meer zijn.

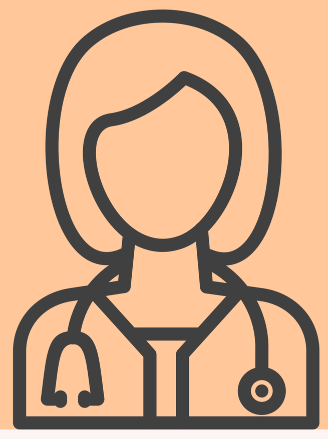

Informeer (tand)artsen voor elke behandeling

Laat alle behandelaren altijd weten dat u een hoger risico heeft op endocarditis. Dit kan er bijvoorbeeld toe leiden dat zij u antibiotica geven voor een behandeling.

Meer informatie over endocarditis?

Bezoek de website van Stichting Pace Makers om meer te lezen over endocarditis en de persoonlijke verhalen van meerdere endocarditis patiënten. Ook de website van de Hartstichting geeft betrouwbare informatie over alle hartziekten.

STICHTING PACE MAKERS

Aandacht voor endocarditis. Aandacht voor leven.

Sla deze patientenkaart digitaal op als PDF

Met uw telefoon als camera kunt u deze QR-code scannen. Als u die link volgt komt u bij een PDF van deze patiëntenkaart. Sla deze op op uw telefoon, zodat u de kaart altijd bij de hand heeft en gemakkelijk aan uw behandelaren kunt laten zien.
